# Spatial variation in prostate cancer survival in the Northern and Yorkshire region of England using Bayesian relative survival smoothing

**DOI:** 10.1038/sj.bjc.6604757

**Published:** 2008-11-04

**Authors:** L Fairley, D Forman, R West, S Manda

**Affiliations:** 1 Northern and Yorkshire Cancer Registry and Information Service, St James's Institute of Oncology, St James's University Hospital, Level 6, Bexley Wing, Beckett Street, Leeds LS9 7TF, UK; 2 Cancer Epidemiology Group, Division of Epidemiology and Biostatistics, University of Leeds, St James's Institute of Oncology, St James's University Hospital, Bexley Wing, Beckett Street, Leeds LS9 7TF, UK; 3 Biostatistics Unit, Division of Epidemiology and Biostatistics, University of Leeds, Worsley Building, Leeds LS2 9JT, UK; 4 Biostatistics Unit, South African Medical Research Council, Private Bag X385, Pretoria 0001, South Africa

**Keywords:** Bayesian analysis, spatial models, relative survival, prostate cancer

## Abstract

Primary Care Trust (PCT) estimates of survival lack robustness as there are small numbers of deaths per year in each area, even when incidence is high. We assess PCT-level spatial variation in prostate cancer survival using Bayesian spatial models of excess mortality. We extracted data on men diagnosed with prostate cancer between 1990 and 1999 from the Northern and Yorkshire Cancer Registry and Information Service database. Models were adjusted for age at diagnosis, period of diagnosis and deprivation. All covariates had a significant association with excess mortality; men from more deprived areas, older age at diagnosis and diagnosed in 1990–1994 had higher excess mortality. The unadjusted relative excess risks (RER) of death by PCT ranged from 0.75 to 1.66. After adjustment, areas of high and low excess mortality were smoothed towards the mean, and the RERs ranged from 0.74 to 1.49. Using Bayesian smoothing techniques to model cancer survival by geographic area offers many advantages over traditional methods; estimates in areas with small populations or low incidence rates are stabilised and shrunk towards local and global risk estimates improving reliability and precision, complex models are easily handled and adjustment for covariates can be made.

Five-year relative survival rates from prostate cancer in England and Wales have increased from 43% for men diagnosed during 1986–1990 to 68% for men diagnosed in 1996–1999. The deprivation gap in survival also increased over this time period to a difference of 7% between men from the most affluent areas compared with men from the most deprived areas (Coleman *et al*, 2004). This increase in survival reflects the increased use of prostate-specific antigen (PSA) testing, which has led to the diagnosis of many asymptomatic prostatic cancers that would never have been diagnosed in life (Brewster *et al*, 2000; Evans and Moller, 2003).

Survival from prostate cancer is known to vary among different geographic regions. The EUROCARE study found that there was considerable variation in survival between countries, with England having one of the lowest survival rates (Post *et al*, 1998; EUROCARE, 2003; Berrino *et al*, 2007). Significant geographical variation in prostate cancer survival has also been observed in Australia (Yu *et al*, 2004), the Nordic Countries (Dickman *et al*, 1997) and the United States (Farrow *et al*, 1996). Within the Northern and Yorkshire regions the 5-year relative survival from prostate cancer has been shown to vary by cancer network from 62 to 72% (NYCRIS, 2004).

Most regional analyses of survival estimate rates for each region separately and compare them to assess the regions that are statistically different from each other. This leads to problems with multiple testing; if there are many areas some will be found to be statistically significantly different through chance alone. In addition, areas with sparse data will have large confidence intervals and will produce unstable estimates of survival rates. Furthermore, independently estimating the survival rates for each area makes no use of data in the surrounding areas. The result is that the area-specific estimates of survival rate have low power and validity, in addition to poor precision.

Most of these problems can be overcome by statistical smoothing techniques, which by borrowing and sharing data across areas, produce more reliable estimates of risk. In particular, estimates are stabilised and shrunk towards average values; thus overcoming the problems of areas with small populations or low incidence rates. Of the many methods available, Bayesian methods are commonly used to generate smoothed estimates of risk in cancer relative survival (Yu *et al*, 2004). Bayesian methods are proving useful and applicable because of the ease with which prior information is included in the analyses; moreover, complex and realistic models are easily handled as opposed to traditional methods. In using a Bayesian approach, we obtain posterior distributions of all the model parameters and their functions such as relative contributions of the unstructured and structured spatial effect variations.

There has been a trend in the last few years within epidemiology to perform spatial variation of diseases risk at the smallest geographical area such as electoral wards. However, in the case of cancer survival where there is a need to calculate expected deaths in follow-up intervals (usually yearly intervals), the observed deaths are very sparse, which makes the assumptions on which estimating methods are based invalid. (Rachet and Coleman, 2004) Cancer survival estimates are not routinely produced at Primary Care Trust (PCT) levels, as PCT level estimates of survival are insufficiently robust. Rachet and Coleman (2004) found that annual estimates of relative survival might not be reliable for PCTs because of small numbers of deaths, even in cancers where the incidence is high. That report was based on the PCTs prior to the most recent reorganisation in October 2006; however, even the newly configured PCTs, although much larger than the previous ones, are still too small to be used as geographic units for monitoring cancer survival. (Ellis *et al*, 2007) Using Bayesian smoothing methods to estimate survival for each PCT will overcome many of these problems by borrowing and sharing data across areas overcoming the problems with areas with low incidence or few deaths. We have used PCT (using pre October 2006 boundaries) as our level of analysis. Moreover PCTs are a logical unit to use as they are the focus of most of the NHS cancer policy and responsible for delivering care to patients and commissioning services within that area. It is also important not only to provide an estimate for each PCT, but also to see how these estimates relate to other PCTs.

We set out to apply full Bayesian smoothing techniques using hierarchical models to assess PCT-level spatial variation in prostate cancer relative survival. By, taking into account both local and global risk smoothing, these methods produce more reliable, precise and robust estimators for PCT-specific variation in prostate cancer relative survival. We also investigated if the spatial variation in survival can be explained by socio-demographic risk factors, age, deprivation and period of diagnosis.

## Materials and methods

### Data

The Northern and Yorkshire Cancer Registry and Information Service (NYCRIS) is a population-based cancer registry covering a total population of 6.7 million (at the 2001 Census). The analysis in this paper is based on the Primary Care Trust boundaries that were effective until October 2006. Within the NYCRIS region there were 44 PCTs during this time period. The average size of the PCT populations in 2001 was just under 150 000, this ranged from just under 70 000 in Eden Valley to about 307 000 in Northumberland.

All prostate cancer (ICD10 C61) cases diagnosed in the NYCRIS region between 1990 and 1999 were extracted from the registry database (*n*=22 042). These cases were then followed up for death until 31 December 2004. There were 492 cases that were Death Certificate Only and no date of diagnosis was available for these cases so they were excluded from the analysis. Cases aged over 100 at diagnosis and cases that were multiple primaries were also excluded from the analysis (2097 cases). This left 19 453 cases to be included in the survival analysis but a further 45 cases had the date of diagnosis the same as the date of death and therefore had zero survival time and were also excluded from the analysis. The resulting study population consisted of 19 408 men diagnosed with prostate cancer between 1990 and 1999 and followed up till the end of 2004.

For each patient we also obtained their age at diagnosis, ward and PCT of residence at diagnosis and year of diagnosis. Age was grouped into four different categories – 15–59 years, 60–69 years, 70–79 years and 80+ years. Deprivation was measured at the ward level and derived from the Index of Multiple Deprivation 2004 Income domain scores (Nobel *et al*, 2004); each patient was assigned a deprivation quintile based on their ward of residence. Year of diagnosis was split into two time periods 1990–1994 and 1995–1999.

### Statistical methods

#### Relative survival

Relative survival is the preferred method of survival analysis for population-based cancer survival. The cause of death is not reliably reported for all cancer patients and even with access to medical records it is difficult to classify each patient's death into one of the two categories ‘entirely due to cancer’ or ‘entirely unrelated to cancer’. Therefore, rather than using cause-specific survival, relative survival is used when calculating survival from cancer. Relative survival is the ratio of the observed proportion surviving in a group of patients to the expected proportion that would have survived in a comparable group of people (with for example, the same distribution by age, sex and the geographical area) from the general population. The major advantage of this measure is that information on cause of death is not required and it provides a measure of the excess death rate experienced by patients diagnosed with cancer, irrespective of whether the excess is directly or indirectly attributable to the cancer (Ederer *et al*, 1961; Hakulinen and Tenkanen, 1987).

#### Modelling relative survival

To model the relative survival so we could include covariates in the analysis we fitted a Poisson model for relative survival as described in Dickman *et al* (2004) and Pohar and Stare, (2006) assuming an additive hazard model. The hazards are assumed to be constant within pre-specified sub intervals of follow-up time (that is, piecewise constant hazards). In this study, we have used follow-up intervals of 1 year. A set of indicator variables was constructed, one indicator variable for each interval, and incorporated into the covariate vector. Our primary interest is in the excess hazard component, which is assumed to be an exponential function of the covariates, written as exp(*zβ*) where z is the vector of predictor variables with the associated vector of coefficients in *β*, that is, the Cox model. Parameters representing the effect in each follow-up time interval are estimated in the same way as parameters representing the effect of, for example, age or sex. In our models we have age at diagnosis (four categories) deprivation quintile (five categories) and period of diagnosis (two categories) as covariates.

We assume that patients are grouped into *k* strata based on a combination of the relevant predictors, that is, one stratum for each combination. Furthermore, assume that there are *i* intervals of follow up. Following Dickman *et al* (2004) let the number of deaths, d_*ki*_, be distributed as a Poisson distribution, d_*ki*_∼Poisson(*μ*_*ki*_) where *μ*_*ki*=_*λ*_*ki*_*y*_*ki*_ and *y*_*ki*_ is person time at risk for the observations in stratum *k* in the interval *i*. If we denote d_ki_^*^ as the expected number of deaths in stratum *k* and interval *i*, then

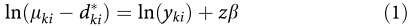


Note that d_ki_^*^ is the expected number of deaths (due to causes other than the cancer of interest and estimated from general population mortality rates). This is a generalised linear model with outcome d_*ki*_ with a Poisson error structure and a link function ln(*μ*_*ki*_−d_ki_^*^) with offset ln(*y*_*ki*_). This is not a standard link function so fitting the model requires software which allows user-defined link functions, in our analysis we fitted it in WinBUGS (Spiegelhalter *et al*, 2004). The exponentiated parameter estimates have an interpretation as excess hazard ratios also known as relative excess risk (RER).

We used the Stata macro strs, written by Paul Dickman (available from http://www.pauldickman.com) to calculate expected survival using the Ederer II method (Dickman *et al*, 2008), using lifetables derived specifically for the Northern and Yorkshire region. Each individual's follow up time was censored at 5 years. For each individual we estimated the expected number of deaths in each follow up interval and also the person time at risk as well as an indicator of whether or not they died in that interval. We then aggregated these values for each PCT for each combination of covariates in each follow up period (4 age groups × 5 deprivation groups × 2 periods of diagnosis=40 combinations of covariate groups (5 follow up periods and 44 PCTs)).

#### Bayesian spatial models

We used Bayesian spatial models to fit both local and global smoothed estimates of relative excess risk across the 44 PCTs (Besag *et al*, 1991). This was done by modifying (1) to include PCT random effects 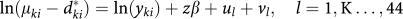 where *u*_1_ and *v*_1_ are the unstructured and structured random effects, which are assigned a normal and a conditional autoregressive prior distribution. The former smoothes the relative risks towards a global value, whereas the latter towards the mean risk of the neighbouring areas, with variance inversely proportional to number of neighbours (Besag *et al*, 1991). Unstructured heterogeneity variance (*σ*^2^) and conditional spatial variance (*θ*^2^) are each given *γ* prior distributions with scale parameter 0.5 and shape parameter 0.001.

### Relative contributions of spatial and unstructured heterogeneity in the convolution model

Unstructured heterogeneity variance (*σ*^2^) and spatial variance (*θ*^2^) are not directly comparable, *σ*^2^ reflects marginal variability of the unstructured random effects between areas whereas, *θ*^2^/*n*_1_ reflects conditional variance of the spatial effect of area *l* conditional on the values of the neighbouring spatial effects, where *n*_1_ is the number of neighbours of area *l*. Therefore, we have to estimate the marginal spatial variance empirically where, 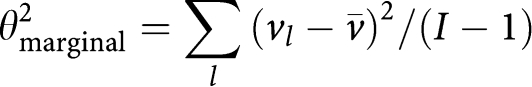


From this we can then estimate the relative contributions of spatial and unstructured heterogeneity. We define the spatial fraction (Frac spatial) as 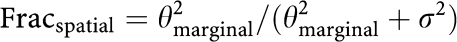


If the spatial fraction is close to 1 then the spatial heterogeneity dominates and if the spatial fraction is close to 0 then the unstructured heterogeneity dominates. Spatially structured variance measures the amount of local smoothing whereas the unstructured variance measures global smoothing in the PCT-relative risks. In the model where possible risk factors are not included, these show the overall variation, otherwise they show excess variation above that captured by the included risk factors. We compared this spatial fraction after including different variables in the model to see how much of the geographical variation between PCTs can be explained by the risk factors.

We fitted four different models, one unadjusted (only including the follow up interval) one adjusted for each covariate in turn and one fully adjusted model, which included all covariates. We used the Deviance Information Criterion (DIC) to asses the model fit (Spiegelhalter *et al*, 2002) where the model with the smaller DIC is better supported by the data. We used a ‘burn-in’ period of 40 000 iterations and then ran a further 40 000 iterations to obtain the posterior estimates. We report the median and 95% credible intervals for the posterior summaries. For comparison we also fitted the models in Stata to obtain RERs for each PCT using a classical Poisson model.

## Results

**Figure 1:**
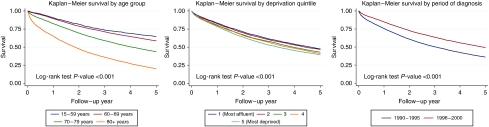
Kaplan–Meier Survival curves by each covariate.

Table 1 shows the distribution of the demographic variables in the study population, with the corresponding 5-year survival and Figure 1 shows the Kaplan–Meier survival curves up to 5 years of follow up by each of the demographic variables. Over 90% of the men were aged 60 and over at diagnosis with 43% of them aged 70–79 years. There were more men from the more deprived areas than the most affluent areas (15% of men were in the most affluent deprivation quintile compared with 24% of men in the most deprived quintile). Slightly more men were diagnosed in the later time period (56%) than in the earlier time period (44%). The overall Kaplan–Meier 5-year survival rate for men diagnosed with prostate cancer in the NYCRIS region was 41% (95% CI 41–42). Survival decreased as age at diagnosis increased; men aged 15–59 years at diagnosis had a 5-year survival rate of 62% whereas it was only 19% for men aged 80 and over at diagnosis. There was a difference of about 8 percentage points in the 5-year survival of men from the most affluent areas compared with those from the most deprived (46 and 38% respectively). The 5-year survival rate of men diagnosed between 1990 and 1994 was significantly lower than for men diagnosed between 1995 and 1999 (35 and 46% respectively).

**Figure 2:**
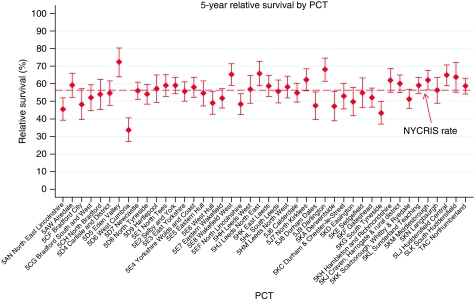
Relative survival by PCT.

The graph in Figure 2 shows the 5-year relative survival rates by PCT. The overall survival rate in the NYCRIS region was 56% (95% CI 55.4–57.3), this ranged from 34% in West Cumbria PCT to 72% in Eden Valley PCT. There were five PCTs that had a significantly higher relative survival rate than the NYCRIS rate and five PCTs that had a significantly lower relative survival rate.

Table 2 shows the results from the Bayesian Poisson models of excess mortality for the unadjusted, univariate and fully adjusted models. The last column also shows the results from the classical Poisson regression and the estimated relative excess risks are very similar to the Bayesian estimates. Comparing the Bayesian models the DIC for the fully adjusted model is about 730 points lower than the unadjusted model and substantially lower than each of the univariate models suggesting that the fully adjusted spatial model is better supported for the data (Table 3). The fixed effects estimates in the univariate and fully adjusted models are similar. From the fully adjusted model the excess mortality associated with a diagnosis of cancer is 2.27 times higher for men aged 80 and over at diagnosis compared with men aged 15–59 years at diagnosis (95% CI 2.02–2.55). There was also significant deprivation effect, with an RER of 1.43 in the most deprived areas compared with the most affluent areas (95% CI 1.28–1.58). Men diagnosed later had a significantly lower excess mortality rate than men diagnosed in the earlier period (RER=0.67, 95% CI 0.64–0.71).

**Figure 3:**
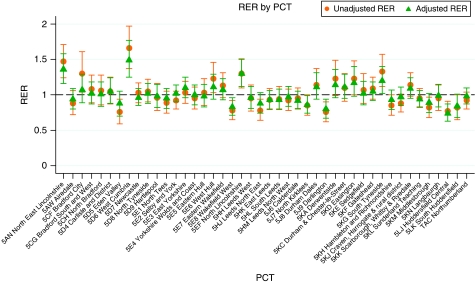
Relative excess risks (RER) by PCT.

Figure 3 shows the unadjusted and fully adjusted RER for each PCT. As expected many of the PCTs with relative survival rates less than the NYCRIS rate had RERs higher than 1 and PCTs with relative survival rates higher than the NYCRIS rates had RERs less than 1. The unadjusted RER range from 0.76 to 1.66; seven PCTs have significantly higher RERs compared with the overall NYCRIS region and seven PCTs have significantly lower RERs. The fully adjusted RERs range from 0.74 to 1.49, and from the graph we can see that the fully adjusted model shrinks the RERs towards the baseline value of 1. The number of PCTs significantly different from the baseline is reduced to four PCTs with significantly higher RERs and four PCTs with significantly lower RERs.

Table 4 shows descriptive statistics comparing the adjusted RERs for the PCTs using a Bayesian and classical approach. As expected under Bayesian hierarchical spatial analysis, the PCT-relative excess risk estimates are less dispersed around the median of 1 than the classical estimates.

In the unadjusted model 70% of the variance between PCTs was due to spatial effects although the 95% credible interval for this was from 2 to 99%. In each of the univariate models the percentage of the variance explained by the spatial effects was similar ranging from 67% for the model adjusted for period of diagnosis to 72% for the model adjusted for deprivation. In the fully adjusted model the fraction of the variation that was spatially structured was 74%, and the width of the credible interval was narrower, from 14 to 98%. The percentage of spatially structured variance in the fully adjusted model was greater than the unadjusted model and the univariate models suggesting that all three covariates might not be truly independent.

**Figure 4:**
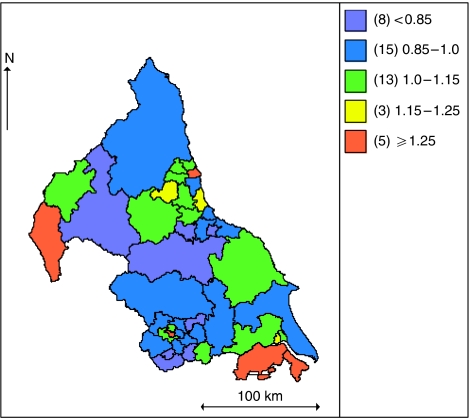
Map of unadjusted smoothed PCT spatial effects.

**Figure 5:**
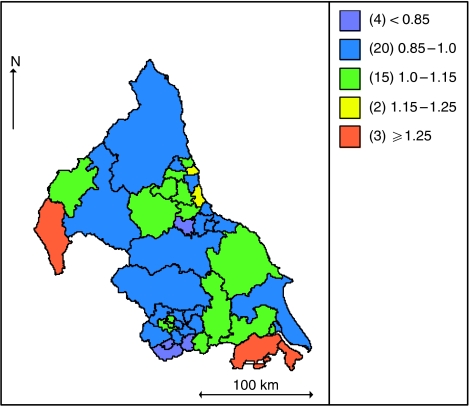
Map of fully adjusted smoothed PCT spatial effects.

The maps in Figures 4 and 5 show the unadjusted and fully adjusted spatially smoothed RERs for each PCT. After adjusting for the covariates areas of high and low excess mortality were smoothed towards the mean, the number of PCTs with RER above 1.25 reduced from five to three whereas the number of PCTs with RER under 0.85 reduced from eight to four. After adjusting for the covariates area with higher excess risk of deaths were found in West Cumbria, North Lincolnshire and North East Lincolnshire whereas the areas with low excess risk of death were found in Huddersfield Central, South Huddersfield, Darlington and Wakefield West.

## Discussion

Many studies have found that there is regional variation in prostate cancer survival (Farrow *et al*, 1996; Dickman *et al*, 1997; Post *et al*, 1998; EUROCARE, 2003; Yu *et al*, 2004; Berrino *et al*, 2007), we also found that there are differences in the survival patterns for PCTs in the NYCRIS region. The estimation of cancer survival at the PCT level can be problematic due to insufficient data to obtain robust estimates. In this paper, we have used Bayesian methods and adjusted for socio-demographic risk factors to produce survival estimates, in the form of relative excess risks of death, by PCT for prostate cancer in the NYCRIS region. By mapping the results we were able to identify the areas where high excess mortality was evident and where resources may be targeted to improve outcomes.

The results showed that all of the three included covariates had a significant association with excess mortality rates, men from more deprived areas, older age at diagnosis and diagnosed between 1990 and 1994 had higher excess mortality. However, the analysis has suggested that there may be some other risk factor that we have not accounted for that influences survival. Both stage of disease at diagnosis and treatment received will have an impact on patient survival.

Cancer stage is one of the most important predictors of survival. In New South Wales, Australia Bayesian models that adjusted for stage of disease were fitted and significant variation in prostate cancer survival between regions was found. (Yu *et al*, 2004) The cancer registry does not have good quality data on stage for the Northern part of the NYCRIS region until the late 1990s, therefore, we are unable to adjust for this in our models and analysis of these variables over our study period is limited. However, some analysis on metastases rates across the PCTs based on data from 1998 and 1999 has been carried out (data not shown). The rate of metastases at presentation by PCT show that the three PCTs with higher excess risk of death had higher percentages of men presenting with metastases than the NCYRIS average whereas the four PCTs with lower excess risks of death had lower percentages of men with metastases. The differences in the proportions of men presenting with metastases across the PCTs may help explain some of the variation in survival that we have observed.

The stage at diagnosis is likely to have changed over time as the use of PSA testing has become more common in the UK (Brewster *et al*, 2000; Evans and Moller, 2003). This increase in PSA testing has lead to the diagnosis of many cancers earlier and that might not have been diagnosed in life. By diagnosing many more tumours earlier survival estimates will increase. We saw in our study the men diagnosed in the later time period had an excess risk of death 33% lower than men diagnosed in the earlier half of our study period.

There is also evidence to suggest that PSA testing is more common in affluent areas (Melia *et al*, 2004). Coleman *et al* (2004) found that in England and Wales inequality in deprivation increased over time and part of this increase in inequality could be because of increased PSA testing in the more affluent groups. There is likely to be a complex relationship between stage and deprivation and survival that is not accounted for in our models.

Treatment received for prostate cancer will also have a bearing on the survival of each individual. Currently in the UK there is no consensus on the best treatment for prostate cancer (NICE, 2002), and there are wide variations in the methods of treatment used in the UK. (Hanna *et al*, 2002; Payne and Gillatt, 2007) Geographical differences in treatment have been observed elsewhere (Bauvin *et al*, 2003; Krupski *et al*, 2005) and in our study region there is some evidence of differences in the treatment of prostate cancer received by cancer network. (NYCRIS, 2007) Looking at treatment data across the PCTs for 1998 and 1999 also shows that there is a wide variation in treatment modality by PCT (data not shown).

This study identified three areas with higher excess risks of death and four areas with lower risks of death after adjustment for age at diagnosis, deprivation and period of diagnosis. The age standardised incidence rates for the PCTs with higher excess risk were lower than the NYCRIS rate in 1990–1994 (one of these was statistically significantly lower) and two of the three PCTs had lower rates than the NYCRIS rate in 1995–1999 (one of these was statistically significantly lower). All the incidence rates for the four PCTs with lower excess risks were higher than the NYCRIS rate in 1990–1994 and this was statistically significantly higher in three. The rates were also higher for these PCTs in 1995–1999 and two of these were statistically significantly higher. The areas with higher rates may be diagnosing more cases due to increased use of PSA testing, therefore increasing survival rates as many of these cases will have early stage disease. Gavin *et al* (2004) found that there was a 100-fold variation in PSA testing rates across general practises in Northern Ireland, we do not have data to assess the variation in levels of PSA testing across PCTs but differences in PSA testing by PCT will influence both incidence rates and survival rates by PCT.

We found that there was a significant deprivation gradient in the excess mortality; men from the most deprived areas had an excess mortality rate that was about 47% higher than that of men from the least deprived areas, this was attenuated slightly to 43% in the fully adjusted model. Deprivation was measured at the ward level, we do not have socio economic information at the individual level so have to use an area-based measure and should be aware that the results obtained for each area cannot be extrapolated to the individuals within that area.

In the fully adjusted model we found that the percentage of spatially structured variance was greater than the unadjusted models and the univariate models. This suggests that all three of the covariates in the model, age, deprivation quintile and period of diagnosis, might not be truly independent. There was a higher percentage of younger men diagnosed in 1995–1999 compared to 1990–1994, and there were more younger men diagnosed in affluent areas compared with the more deprived areas (data not shown).

We compared this spatial fraction after including different variables in the model to see how much of the geographical variation between PCTs can be explained by the risk factors. In the fully adjusted model 74% of the variation in survival is spatially structured which may indicate that there are some other spatially structured risk factors that we have not accounted for that may help explain the variation between the PCTs, for example differences in treatment or stage at diagnosis by PCT.

Many spatial analysis of incidence and mortality use data at a smaller geographical level than PCT, maybe ward or output area. Jarup *et al* (2002) used Bayesian methods to analyse spatial variations in prostate cancer incidence by ward in Great Britain and found there was no marked geographical variation in the risk of prostate cancer; however, this study used data from 1975 to 1991 before the use of PSA testing was more wide spread in the UK. This unit of analysis is not suitable for survival analysis as the models require a certain number of cases/deaths per interval. Also as treatment may influence survival and many patients will be treated within the same PCT, the PCT is a more logical geographic unit to use in survival analysis. However, we did carry a similar analysis based on ward of residence and found similar results as when we used the PCT data. There were clusters of wards with the highest excess risks of death in Cumbria, North Lincolnshire and North East Lincolnshire.

Bayesian methods are more frequently used to study spatial variation in cancer survival (Osnes and Aalen, 1999; Henderson *et al*, 2002; Yu *et al*, 2004) The use of Bayesian techniques to model cancer survival offers many advantages over the traditional methods. In particular, risk estimates in areas with small populations or low incidence rates, especially rural areas, are stabilised and shrunk towards local and global risk estimates. This improves the reliability and precision of the risk estimates. Therefore the chance of obtaining excessively high or low estimates due to the occurrence of sparse data is reduced. Other advantages of using a Bayesian approach are that it is relatively easy to fit complex models that allow adjustment for covariates when assessing geographical variation in survival and we can also obtain functions of the model parameters such as the relative contributions of the unstructured and structured spatial effects.

In our models we used an adjacency matrix that was based on the number of neighbours each PCT had. We used all of the PCTs in the NYCRIS region and some of these PCTs will border PCTs that are in a different cancer registry. In our study, these PCTs will only have recorded the PCTs that are in the NYCRIS region and this may introduce some bias into the results as these areas have neighbours missing. The credible interval for the estimates of the amount of variation that is due to spatially structured effects was rather wide; ranging from 2 to 99% in the unadjusted model, this decreased slightly in the fully adjusted model ranging from 14 to 98%. The marginal spatial variance is inversely related to the total number of areas and in this study we only have 44 areas, the conditional spatial variance is also inversely proportional to the number of neighbours each area has and in this study we used quite large areas and they had relatively few neighbours, therefore the precision of the variance estimates would be smaller if we had used more smaller areas with more neighbours (such as wards).

The ability to detect areas where cancer survival is poor is a useful tool as it indicates where the policy to improve survival might be best targeted or where further investigation to understand the causes of the poor survival might be best carried out. As in this study it is important to be able to adjust for some common confounding variables such as age and deprivation. We feel that this methodology could be useful to apply to other cancer sites and geographic areas to gain a better understanding of regional variation in survival.

## Figures and Tables

**Table 1:** Demographic characteristics of the study population and Kaplan–Meier 5-year survival rates

**Variable**	***N***	**%**	**5-year Kaplan–Meier survival (%)**	**95% CI**
All cases	**19 408**		41.2	(40.5, 41.9)
				
*Age group*				
15–59 years	1123	5.79	62.4	(59.5, 0.65.2)
60–69 years	4920	25.35	56.8	(55.4, 58.1)
70–79 years	8432	43.45	42.2	(41.1, 43.2)
80+ years	4933	25.42	19.3	(18.2, 20.4)
				
*Deprivation quintile*				
1 (most affluent)	2809	14.47	45.5	(43.7, 47.3)
2	3612	18.61	45.1	(43.5, 46.7)
3	3662	18.87	41.1	(39.5, 42.7)
4	4638	23.90	39.3	(37.9, 40.7)
5 (most deprived)	4687	24.15	37.7	(36.3, 39.0)
				
*Period of diagnosis*				
1990–1995	8556	44.08	35.0	(34.0, 36.0)
1996–2000	10 852	55.92	46.1	(45.2, 47.1)

**Table 2:** Covariate fixed effects of estimates of the relative excess risk of death

		**Bayesian**				**Classical**
**Variable**	**Factor**	**Adjusted for age**	**Adjusted for deprivation**	**Adjusted for period**	**Adjusted for age, deprivation and period**	**Adjusted for age, deprivation and period**
*Fixed effects*						
Age group	15–59 years	1.0			1.0	1.0
	60–69 years	0.99 (0.88, 1.12)			0.99 (0.88, 1.12)	0.99 (0.88, 1.12)
	70–79 years	1.29 (1.15, 1.44)			1.28 (1.14, 1.43)	1.27 (1.13, 1.42)
	80+ years	2.34 (2.08, 2.64)			2.27 (2.02, 2.55)	2.23 (1.98, 2.51)
Deprivation quintile	1 (most affluent)		1.0		1.0	1.0
	2		0.99 (0.88, 1.12)		0.98 (0.88, 1.09)	0.97 (0.87, 1.08)
	3		1.23 (1.09, 1.37)		1.21 (1.08, 1.34)	1.21 (1.09, 1.34)
	4		1.30 (1.16, 1.46)		1.27 (1.14, 1.41)	1.30 (1.18, 1.44)
	5 (most deprived)		1.47 (1.31, 1.64)		1.43 (1.28, 1.58)	1.45 (1.32, 1.60)
Period of diagnosis	1990–1995			1.0	1.0	1.0
	1996–2000			0.64 (0.60, 0.68)	0.67 (0.64, 0.71)	0.67 (0.63, 0.71)

**Table 3:** Variance estimates for the random effects

**Variance**	**Unadjusted**	**Adjusted for age only**	**Adjusted for deprivation only**	**Adjusted for period only**	**Adjusted for age, deprivation and period**
*PCT*					
Unstructured	0.0124 (0.0005, 0.0476)	0.0113 (0.0005, 0.0420)	0.0083 (0.0004, 0.0340)	0.0131 (0.0005, 0.0472)	0.0064 (0.0005, 0.0241)
Structured	0.0286 (0.0010, 0.0510)	0.0245 (0.0013, 0.0452)	0.0218 (0.0010, 0.0395)	0.0269 (0.0015, 0.0502)	0.0177 (0.003, 0.032)
Fraction structured	0.70 (0.02, 0.99)	0.69 (0.04, 0.99)	0.72 (0.03, 0.99)	0.67 (0.04, 0.99)	0.74 (0.14, 0.98)
DIC	19 464.8	18 996.1	19 400.4	19 236.2	18 738

**Table 4:** Descriptive statistics comparing adjusted RER for PCTs from Bayesian spatial and classical methods

**Measure**	**Bayesian RER**	**Classical RER**
Min	0.74	0.44
25th centile	0.93	0.8
Median	0.99	0.99
75th centile	1.08	1.31
Max	1.49	1.68
Number of PCTs with RER <0.85	4	14
Number of PCTs with RER >1.25	3	12
